# Expression pattern of the GRAS gene family during somatic embryogenesis in pine

**DOI:** 10.1186/1753-6561-5-S7-P136

**Published:** 2011-09-13

**Authors:** Inmaculada Hernández, Elena Carneros, Alberto Pizarro, Dolores Abarca, Carmen Díaz-Sala

**Affiliations:** 1 Department of Plant Biology, University of Alcalá, Alcalá de Henares, Madrid, 28871, Spain; 2 Department of Plant Biology, Universtiy of Alcalá, Alcalá de Henares, Madrid, 28871, Spain

## 

The GRAS protein family of putative transcription factors, which includes SHORT-ROOT (SHR), SCARECROW (SCR) and SCARECROW-LIKE (SCL) proteins, is involved in root development in *Arabidopsisthaliana* and other plant species [1]. In forest species, genes with homology to the *A. thalianaSCR* gene have been involved in the induction of somatic embryogenesis in *Picea glauca* (Moench) Voss [2] and *Pinus taeda* L. [3] as well as in the development of radial patterning of roots in *Pinus sylvestris* L. [4]. Schrader *et al*[5] also reported the expression of genes with homology to the *A. thalianaSHR* gene in cambial region of *Populus tremula x tremuloides*. Increased levels of mRNA of *Pinus radiata SHR* (*PrSHR*), *Pinus radiata SCARECROW-LIKE1* (*PrSCL1*) and *Castanea sativaSCARECROW-LIKE1* (*CsSCL1*) have been associated with the early stages of adventitious root induction in *Pinus radiata* D. Don and *Castanea sativa* Mill., respectively [6-9].

In addition to *PrSHR* and *PrSCL1*, we have identified 13 new *GRAS* genes belonging to the different *GRAS* clades in the pine genome. The objective of this work is the analysis of the spatiotemporal expression patterns of the pine *GRAS* gene family during somatic embryogenesis in *Pinus radiata* D. Don. Somatic embryogenesis has become the first biotechnology showing great potential for mass propagation of conifers for application in forestry, allowing the implementation of multivarietal forestry (MVF) [10,11]. Despite major advances in clonal regeneration by somatic embryogenesis or organogenesis, many forestry species are recalcitrant [12]. More knowledge of the regeneration process regulation is necessary to improve the capacity of vegetative regeneration.

The expression pattern of the genes was analyzed by qRT-PCR following the methodology described by Sánchez *et al*[6] and Solé *et al*[7]. For expression analysis, total RNA was extracted from four stages of the somatic embryogenic process: proliferative tissue after 7 and 14 days from the last transference to proliferation medium, somatic embryos at the beginning of differentiation and cotyledonary somatic embryos.

In general, the transcripts of the pine *GRAS* genes accumulated at the highest levels in cotyledonary somatic embryos. In addition, the transcript levels of *PrSCR*, *PrSHR*, *PrSCL1*, *PrSCL6*, *PrSCL8*, *PrSCL11* and*PrSCL12* showed an increase in somatic embryos at the beginning of differentiation. No differences in *PrSCL10* transcript levels were found between the four stages analyzed. Transcript levels of *PrSCL16* were undetectable at all stages. *In situ* hybridization for spatial expression analysis will confirm differential expression domains.

This work has been funded by the Spanish Ministry of Science and Innovation (AGL-2008-05105-C02-01/FOR).

Embryogenic lines were provided by C. Walter (Scion).
